# Varicella‐zoster virus in actively spreading segmental vitiligo skin: Pathological, immunochemical, and ultrastructural findings (a first and preliminary study)

**DOI:** 10.1111/pcmr.13064

**Published:** 2022-10-09

**Authors:** Yvon Gauthier, Sebastien Lepreux, Muriel Cario‐Andre, Jérome Rambert, Adrien Dakdaki, Marie‐Edith Lafon, Redouane Abouqal, Laila Benzekri

**Affiliations:** ^1^ Vitiligo and Melasma Research Association (V.M.R.A.) Bordeaux France; ^2^ Pathology Department Hopital Robert Boulin, CHR Libourne France; ^3^ Bordeaux University, INSERM, BRIC, U1312 Bordeaux France; ^4^ National Reference Center for Rare skin Diseases Bordeaux University Hospital Bordeaux France; ^5^ Aquiderm Bordeaux France; ^6^ Tumor Bank and Tumor Biology Laboratory CHU Bordeaux Pessac France; ^7^ Department of Virology Bordeaux University Hospital Bordeaux University, CNRS, UMR5234 Bordeaux France; ^8^ Laboratory of Biostatistics, Clinical Research and Epidemiology Mohammed V University in Rabat, Acute Medical Unit, Ibn Sina Teaching Hospital Rabat Morocco; ^9^ Dermatology Department, Ibn Sina Teaching Hospital Mohammed V University in Rabat, Pigmentary Disorders Outpatient Clinic Rabat Morocco

**Keywords:** autonomic nervous system, melanosome transport, nucleus fusion, segmental vitiligo, syncytium, ultrastructural study, varicella‐zoster virus, viral cytopathic changes

## Abstract

Segmental vitiligo (SV) is a unilateral subtype of vitiligo which is clinically characterized by a cutaneous depigmentation and histologically by a melanocyte loss from the epidermis and hair follicle reservoirs. To date, its pathogenesis remains a mystery. In many cases, this skin depigmentation shares several clinical features and dysfunctions with herpes zoster (HZ). So, for the first time, we examined whether any nucleus and cell fusion associated with a positive immunolabelling of varicella‐zoster virus (VZV) and VZV mature virions could be found in SV skin samples as in herpes zoster (HZ). A total of 40 SV samples were used for histological and immunochemical studies. Control samples were obtained from three HZ, and 10 generalized vitiligo lesions. For ultrastructural study, three recent SV and one HZ as controls were recruited. Here, we report that nuclear fusion in epidermal cells were statistically associated with recent SV (*p* < .001), whereas syncytia formation was associated with long‐lasting SV (*p* = .001). A positive detection of VZV antigen was statistically associated in the epidermis with recent SV and in the dermis with long‐lasting SV (*p* = .001). Finally, the discovery of mature virions in 3/3 recent SV samples provides additional arguments for our viral hypothesis.

**Significance**

A possible role of VZV in SV pathogenesis could be evoked following the concomitant discovery in the same melanocytes of characteristic mature VZV virions, perinuclear clustering, and lysis of mature melanosomes. These abnormal distribution and degradation of melanosomes were previously reported in several lysosomal diseases due to various gene mutations and were attributed to a defective transport and transfer of mature melanosomes to keratinocytes. Therefore, in SV, an inhibition of normal melanosome trafficking in melanocytes following a local virus infestation could be the first step of a cascade of events leading to a depigmentation and possibly to an autoimmune process.

## 1 INTRODUCTION

Segmental vitiligo (SV) is a subtype of vitiligo characterized by a progressive and unilateral cutaneous depigmentation due to a melanocyte loss from both epidermis and hair follicle reservoirs (Ezzedine, Diallo, et al., 2012; Ezzedine, Lim, et al., 2012). About 10% of vitiligo cases are segmental (SV). In these cases, after an initial rapid spreading, the affected area of the skin does not expand with time. Until now, the exact pathophysiology of SV remained unclear. Histological and immunological studies were sparse and mainly focused on the absence of melanocytes and the analysis of lymphocytic infiltration in early evolving SV and mixed vitiligo (Attili & Attili, 2013; Shin et al., 2016; Xu et al., 2021). Few conflicting but not incompatible theories (van Geel et al., 2011) were mainly proposed for SV pathogenesis, such as neural mechanism (Arnozan & Lenoir, 1922; Gauthier & Benzekri, 2010; Koga, 1997), somatic mosaicism (Attili & Attili, 2015; Ezzedine et al., 2011; Taïeb et al., 2008; van Geel et al., 2013), adhesivity impairment (Grill et al., 2018), autoimmunity (Speeckaert et al., 2020), and microvascular skin homing (Ogg et al., 1998). A viral origin was previously evoked mainly in non‐segmental vitiligo (NSV), but was not histologically demonstrated (Dwiwedi et al., 2018; Grimes et al., 1996; Iverson, 2000). However, a possible role of VZV in SV pathogenesis could be suspected, taking into account its possible tropism for pigment cells (Harson & Grose, 1995) and numerous clinical and physio‐pathological similarities with herpes zoster (HZ).

Among the similarities shared between SV and HZ (a good model of VZV reactivation), we can mention the following features:

1. frequent and similar quasi‐dermatomal or Blashkoid distribution (Gyula, 1982; Kwon et al., 2018) with unilateral or sometimes bilateral lesions (Kantari, 2015; Lee & Hann, 1998);
2. some common physiological abnormalities in skin such as an increased blood flow (Wu et al., 2000) and a sweating impairment (Ushigome et al., 2017);
3. a possible but rare occurrence of SV in the same location of the previous HZ (Kim et al., 2018) and, on the contrary, the onset of HZ over the same dermatome of preexisting SV (Gambhir et al., 2014); and
4. a good repigmentation following anti‐VZV treatments with valacyclovir reported in two cases of SV (Abdelmaksoud et al., 2019; Lewis et al., 2018).

Varicella‐zoster virus is a ubiquitous human alpha‐herpesvirus that infects and becomes latent not only in the cranial and spinal sensory and enteric ganglia but, also, in autonomic ganglia during viremia with varicella. VZV can commonly reactivate in sensory ganglia many years after primary infection to cause HZ (Strauss, 1994), but it can also reactivate in autonomic sympathetic ganglia or enteric nervous system, causing various vascular or digestive diseases without any rash (Gershon & Gershon, 2018; Gilden & Nagel, 2016). In all cases, the spread of VZV is histologically associated with characteristic cytopathic changes of the target organ, including the extensive fusion of cell and nuclear membranes leading to the formation of multinucleated syncytia (Knutton, 1978; Leroy et al., 2020; Mueller, 2008; Wang et al., 2017). Cell fusion seems to be a mechanism facilitating viral spreading from infected to uninfected cells. Cytopathic changes are considered the hallmark of VZV pathology and can be retrospectively demonstrated.

In zoster without rash (zoster sine herpete; Zhou et al., 2020), VZV implication could be retrospectively suspected using histology successively to detect characteristic cytopathic effects (Knutton, 1978) and immunochemistry (anti‐VZV antibody) of skin samples (Muraki et al., 1992; Nikkels et al., 1995). The best confirmation of an infestation is provided through the detection of VZV DNA and the visualization of characteristic mature herpes “viridae” virions (180‐200 nm in diameter) by transmission electron microscope (TEM; Lutzner, 1962). The VZV DNA detection in skin with PCR (Polymerase Chain Reaction) and the assessment of VZV IgM could be performed only for a short time after the onset of the depigmentation. In HZ, the duration of the detection of VZV IgM antibodies in the serum was in mean 3.5 weeks after the onset of the rash (Min et al., 2016). So, the assessment of VZV IgM is not useful tool for diagnosis.

This data lead us to examine, whether viral cytopathic changes associated to a positive immunolabelling against VZV antigen and the presence of characteristic mature virions can be concomitantly found in pigmented margins of recent SV as in HZ lesions. Forty patients without a recent HZ and VZV history, mean age 29.6 ± 16.1, diagnosed by two vitiligo experts in the Dermatology Department of Ibn Sina Hospital University Rabat and 18 controls, including 10 controls with recent generalized vitiligo (GV), mean age 30.4 ± 10.5 SD, three herpes zoster, and five healthy skin samples, mean age 28.3 ± 11 SD were recruited from August 2019 to January 2021 (Table 1). High titers of VZV IgG antibodies were found in the serum with chemiluminescence immunoassay (C.L.I.A) and enzyme‐linked immunosorbent assay (E.L.I.S.A), (Table S1): With CLIA technique: titer range was (from 554 to 3174), positivity (>175), and median (943); with ELISA technique, titer range was (from 2.6 to 10), positivity (>1.2), and median (4.5).

**TABLE 1:** Clinical characteristics of patients investigated in the study

Diseases	No. of subjects	Mean age	Sex ratio	Distribution	Onset < 4 months	Onset > 4 months	Localization
Segmental Vitiligo	40	29.6 ± 16.1	M/F: 0.74	Unilateral 52.5% D 47.5% N.D	13	27	Facial: 27 Ex.facial: 13
Generalized. vitiligo	10	30.4 ± 10.5	M/F: 0.25	Bilateral	–	10	Facial: 2 Ex. Facial: 8
Controls	5	28.3 ± 11	5F	–	–	–	Chest: 5
Herpes zoster	3	52	3M	Unilateral	3	–	Facial: 2 Trunk: 1

Abbreviations: F, Female; M, Male.

**FIGURE 1:**
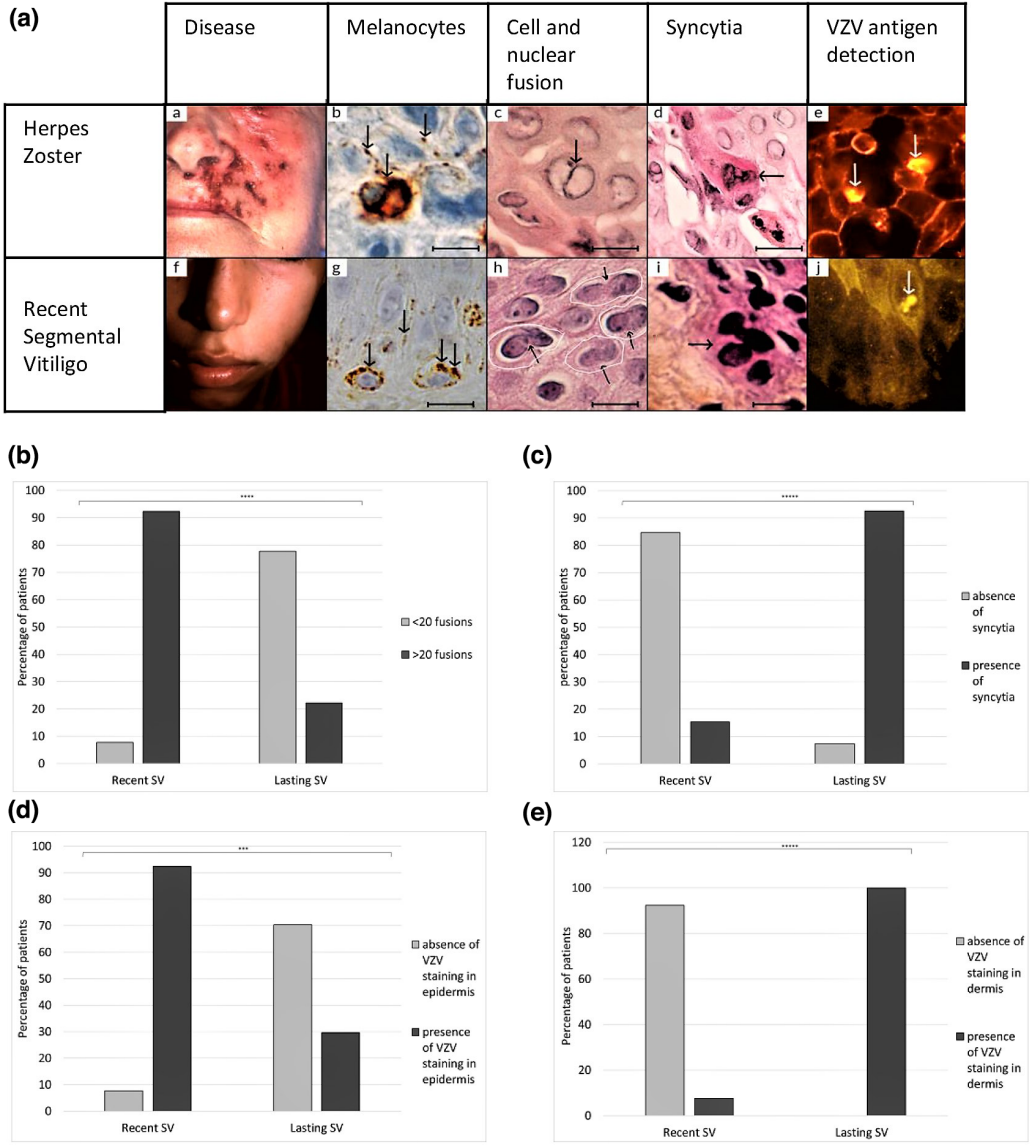
Histological and immunochemical changes in HZ and in pigmented SV skin. (a) Herpes zoster: *Panel a*, HZ of left cheek; *Panel b*, a melanocyte heavily pigmented with large melanin granules in the dendrites (arrow) detected by immunohistochemistry using anti‐HMB45 revealed with DAB in brown, ×40; *Panels c and d* hematoxylin–eosin‐safran staining showing cell fusion between 2 keratinocytes (arrow), ×40 (c), and a giant polynucleated cell, (arrow) ×40 (d); *Panel e*, important VZV immunolabelling in epidermal cells (anti VZV antibody revealed using alexa 555 coupled secondary antibody) (white arrows), ×40 IF. Segmental vitiligo: *Panel f*, recent SV in left cheek; *Panel g*, marginal melanocytes with several melanin giant granules around the nucleus and within dendrites (arrows), detected by immunohistochemistry using anti‐HMB45 revealed with DAB in brown ×40; *Panels h and i* Hematoxylin–eosin‐saffron staining showing nuclear fusion between several keratinocytes (black arrows, surrounded in white), ×40 (h) and a large syncytia rarely found in the epidermis (arrow), ×40 (i); *Panel J*, VZV immunolabeling in an keratinocyte (anti VZV antibody revealed using alexa 555 coupled secondary antibody) (white arrow), ×40. (b) Percentage of SV patients with nuclear fusion (<20 or >20) in epidermis according to the age of SV lesions. (c) Percentage of SV patients without or with syncytia in skin according to the age of the lesions. (d) Percentage of SV patients with negative or positive VZV staining in epidermis according to the age of SV lesions. (e) Percentage of SV patients with negative or positive VZV staining in dermis according to the age of SV lesions. All quantifications were performed by section in recent (*n* = 13) and long‐lasting vitiligo (*n* = 27) patients by two blind observers.

By immunohistochemistry anti HMB‐45 or anti MITF (BSB‐3693, BSB 3657; Bio SB, Santa Barbara, CA 93111 USA), no melanocyte was found in the depigmented areas of SV patients. Whereas, in the pigmented edge of recent SV (*n* = 13; onset of the disease ≤4 months), melanocytes appeared heavily pigmented around the nucleus and sometimes loaded with giant melanin granules in the cytoplasm and within the dendrites (Figure 1A). Very few dendritic expansions were seen in the intercellular space. Keratinocytes were usually hypopigmented except those in direct contact with melanocytes (Figure 1A). In long‐lasting SV (*n* = 27 onset of the disease>4 months), marginal melanocytes with cell or nuclear fusion were seen (Figure S1). Additionally, damages to the sweat glands and the superficial nerves (stained with anti‐NGFR 5BSB‐6291, BioSB) were frequently associated with a discrete lymphocyte infiltrate, which was not investigated (Figures S2 and S3). We graded the magnitude of the cytoplasmic and nuclear membrane fusion of keratinocytes or melanocytes in each section as follows: 0 (i.e., no fusion), 1 (i.e., <20 cell or nuclear fusion), or 2 (i.e., >20 cell or nuclei fusions). The main significant feature observed in recent SV was nuclear fusion in epidermis since 92% of patients with recent SV presented more than 20 nuclei fusions of keratinocytes and melanocytes/section against 22% in long‐lasting SV (Fischer's exact test *p* = 3.4E‐5) (Figure 1A,B). In long‐lasting SV, the main characteristic was the presence of syncytia in dermis mainly located in sweat and sebaceous glands, hair follicles, and vessels since 92% of patients with long‐lasting SV presented syncytia against 15% in recent vitiligo (Fischer's exact test *p* = 2.3E‐6), (Figure 1A,C; Figure S4). In the controls, these investigations were negative, multinucleated cells and syncytia were absent in GV and healthy controls.

A strong positivity of VZV immunolabelling (anti‐VZV BSB2295; BioSB) was detected in all three HZ skin samples (Figure 1A). Anti‐VZV antibody is a cocktail of seven mouse monoclonal antibodies clone SG‐1, SG1‐SG4, NCP1, and IE‐62. As for nuclear fusion and syncytia, VZV staining differed between recent and long‐lasting vitiligo. In recent SV, a slight VZV staining in epidermal cells was observed in 92% of patients, whereas it was only observed in 30% of long‐lasting SV (30%) (Fischer's exact test *p* = .0004) (Figure 1A,D). In recent SV, VZV staining was also noted in some superficial nerves (Figure S3). On the contrary, VZV staining was found, in dermal macrophages and in adnexal cells in all long‐lasting SV, whereas it was observed only in 8% of recent SV (Fischer's exact test *p* = 2.3E‐9) (Figure 1E). These investigations were negative in the controls (GV, healthy and skin samples). There is a positive correlation between epidermal VZV staining and epidermal fusion (ρ: 0.804 CI 95% [0.6571; 0.8921], *p*‐value: 4.153E‐10), and between syncytia and VZV dermal staining (ρ: 0.7507CI 95% [0.5732, 0.8609], *p*‐value: 2.423E‐8).

Histologically and immunochemically, the expression of cytopathic changes and immunolabelling against VZV appears depending on the age of SV lesions. So, at the initial period, the perinuclear clustering of melanosomes could correspond to a positive immunolabelling by early viral structural (IE 63) and late proteins in some epidermal cells (Nikkels et al., 1995). Later, in long‐lasting SV, the formation of syncytia was probably linked with the residual immunoreactivity of the envelope glycoproteins E and H, which remained in the absence of virus for a long time in dermal cells (Cole & Grose, 2003; Pasieka et al., 2004). The mechanism of the fusion of cytoplasmic or nuclear membranes of melanocytes is unclear. A direct effect of VZV on cell cycle regulatory pathways could be discussed (Sattentau, 2008).

**FIGURE 2:**
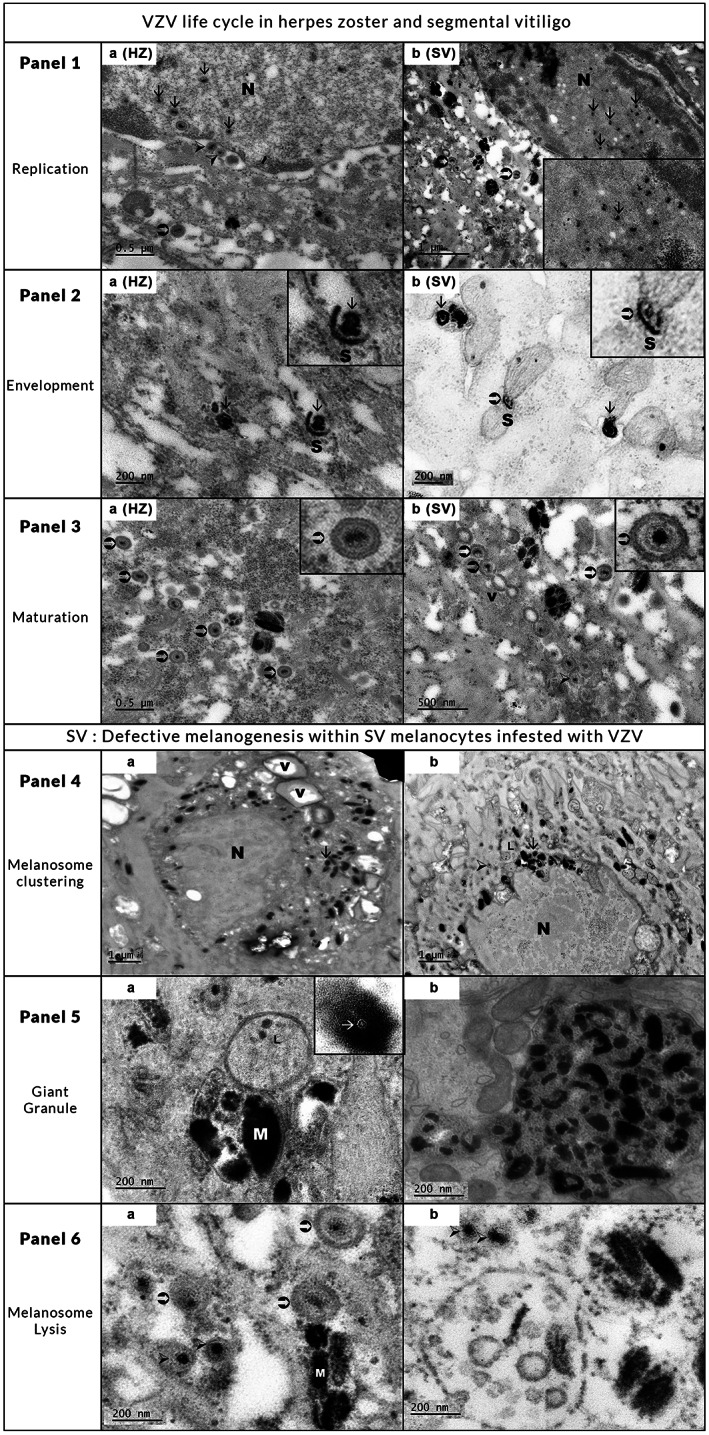
Ultrastructural study of VZV life cycle in HZ and SV and defective melanogenesis in marginal melanocytes of SV. Panel 1: Replication and virus assembly in HZ and SV. (a) VZV capsids (black arrows) in the nucleus, several nucleo capsids (arrowheads) in the nucleus are egressing from the nucleus (N) to perinuclear cisternae, mature virions (white arrow) in the cytoplasm (encart) exit of nucleocapsids through nucleus membrane. (b) several nucleo capsids (black arrows) in nucleus and mature virion (white arrow) in the cytoplasm (encart) nucleocapsids (nc) in the nucleus. Panel 2: Envelopment of nucleocapsids in HZ and SV. (a) (encart) Nucleocapsid (black arrow) adheres to the concave surface of the electron dense sac (S) from Golgi apparatus during the envelopment. (b) envelopment of nucleocapsids (black arrow), (encart) envelopment of nucleo capsid (arrow heads) with electron dense sac (S). Panel 3: Maturation of virions. (a) Numerous nucleocapsids (arrow heads) acquire a characteristic secondary lipid envelopment and become mature virions (white arrows). Encart: a typical VZV mature virion with a nucleo capsid enclosed in a double layer lipid envelope. (b) Several similar mature virions (white arrows) and nucleocapsids (arrow heads) and vesicules (v) in the cytoplasm. Encart: similar aspect of a mature virion. Defective melanogenesis in SV melanocytes infected with VZV. Panel 4: Perinuclear clustering of mature melanosomes and lysosomes. (a) a marginal melanocyte loaded with melanin with a perinuclear clustering of mature melanosomes (black arrow), « honey combed » aspect of the cytoplasm with many large vacuoles (V) Nucleus (N). (b) a perinuclear clustering of large and normal sized melanosomes (black arrow) and lysosomes (L) close to nucleocapsids (arrow heads). Panel 5: Giant melanin granule (a) aggregation of normal and large melanosomes (M), close by a large lysosome (L), encart nucleocapsid (white arrow) in melanin granule. (b) abnormal granule results of the fusion of large and normal sized melanosomes. Panel 6: Lysis of melanosomes. (a) aggregation of several degraded melanosomes (m) close by mature virions (white arrows) and nucleocapsids (arrow heads) (b) a large autophagic vacuole containing residual bodies nearby nucleocapsids (arrow heads) and aggregated melanosomes.

Faced with these interesting data, a search of mature virions in marginal skin of actively spreading SV appeared to be crucial for testing or not our hypothesis. For this purpose, we performed transmission electron microscopy (TEM) on 3 SV samples. In one HZ sample, used as VZV infection control, abundant virus particles including capsids, nucleo‐capsids, and characteristic VZV mature virions were easily detected by transmission electron microscopy (Figure 2 panels 1a, 2a, and 3a). For the first time, similar virus particles, in a lower amount, were found in 3/3 actively spreading SV samples. Among the three skin samples investigated, one was taken from a recent new SV and the two others from preexisting SV, which actively spread 3 weeks ago. The virus particles were more numerous in one case of preexisting SV and were scattered in the nucleus and cytoplasm of a few melanocytes in two other less active cases. Moreover, all steps of the VZV life cycle found in HZ were detected both in HZ and the 3 SV, such as in Figure 2: virus replication within the nucleus and assembly of newly synthetized VZV, envelopment of nucleocapsids, and maturation of characteristic virions. At a higher magnification, we visualized in some marginal melanocytes of SV the characteristic fine structure of some mature virions, including a double layered and electron dense envelope, a tegument, and a nucleocapsid with or without a central core protein. These mature virions were rarely seen in keratinocytes.

Surprisingly, an unusually high number of mature Type IV melanosomes were detected in these infested melanocytes as shown in Figure 2. They exhibited an abnormal and concentrated perinuclear distribution in association with many pre‐melanosomes and lysosomes. A few giant pigment granules previously observed with light microscopy were found. In autophagic vacuoles, several melanosomes that were slightly increased in size were shown to be successively aggregated and degraded. Rare and short dendrites expansions were sometimes filled with degenerative large melanosomes and lysosomes (not shown). In the majority of cases, the keratinocytes were hypopigmented, but when a few abnormal pigment granules were transferred to the keratinocytes they were located in “lysosome like structures” that often surrounded the nucleus (Figure S5).

With TEM, the discovery of pleiomorphic viral particles within a few marginal melanocytes at different steps of virus life cycle attested to the reality of an actual infestation in SV. Moreover, the visualization of characteristic mature virions in 3/3 skin samples provided some additional arguments to our hypothesis. Mature VZV virions were more numerous in one case of actively spreading SV and less abundant in two less extensive SV. The viruses did not infest all the melanocytes observed in the pigmented margin. So, it appears that the successful discovery of viral particles in marginal melanocytes seems to be strongly dependent both on the precocity of the biopsy time and on the extensive rate of SV. After VZV replication and maturation, a low rate of viral particles and rarely of mature virions could be expelled through very few dendrite expansions from the melanocytes to some neighboring keratinocytes.

Concomitantly, an analysis of the defective pigmentation was performed. We noted that the abnormal melanogenesis and the honey‐combed aspect of the cytoplasm were exclusively observed in melanocytes that were infested by VZV. An explanation for this defective melanogenesis can be provided by taking in account the data of previous studies devoted to organelles and melanosomes motility and to organelle diseases. Firstly, in vitro, VZV was suspected to hijack the melanocyte cell machinery for probably supporting its replication and spread, causing a severe dysfunction of melanosome transport and transfer (Barra & Seabra, 2004; Glingston et al., 2019; Harson & Grose, 1995; Tognarelli et al., 2021). In the other part, perinuclear clustering of melanosomes, giant granules development, and scarcity of dendrite expansions were previously found in some organelles diseases such as Chediak Higashi (CHS) and in beige mice (Provance et al., 1996). In this disease, the hypopigmentation associated with severe immunologic defects, is attributed to an intracytoplasmic defective transport and transfer of melanosomes caused by mutation of the LYST gene (lysosomal trafficking regulator; Introne et al., 1999; Shiflett et al., 2002). Consequently, in SV, the hijacking of the melanocyte cellular machinery by VZV could be possibly responsible for a defect of intracellular melanosome transport and a failure of their transfer to keratinocytes. The numerous and large autophagic vacuoles seen in cytoplasm could be likely considered as a melanocytic self‐defense mechanism (Setahuri, 2015; Tognarelli et al., 2021).

Several limitations existed in this preliminary study. The main limitation was the small number of patients recruited either for the ultrastructural or immunochemical and histological studies from a single center of study. Secondly, the virus reactivation could presumably start several days or weeks before the onset of a visible depigmentation. So, according to the time of the biopsy, the cytopathic changes could coexist or not for a short time with a positive VZV immunostaining in the epidermis.

Finally, the data of this preliminary study could strongly suggest the role of VZV in the pathogenesis of SV. Clinically, it seems that the complications due to a brief VZV reactivation might not be restricted to the induction of HZ. VZV reactivation can occur in autonomic or enteric ganglia inducing some localized damages with or without rash. The mean age of SV patients was 29.6 years, while HZ commonly occurred in older patients. So, it could be hypothesized that in some immunocompetent and young people, variable acute stressors, not linked to the age could downregulate some functions of specific immunity triggering a VZV reactivation. The fact that VZV does not spread outside innervated areas likely implies that host immunity halts cell to cell spread except in infrequent mixed vitiligo, which associates SV to generalized Vitiligo.

We hypothesize that in SV, an occult, brief and non‐contagious VZV reactivation could be the first step of a cascade of events leading to a local depigmentation, and possibly to an autoimmune process. In this hypothesis, we could consider SV as a chronic depigmented sequelae developed in a localized area, following unique or successive viral reactivations of brief duration and low‐grade intensity occurring in anatomically corresponding autonomic ganglia.

## AUTHOR CONTRIBUTIONS

**Y. Gauthier** (*Dermatologist*) was involved in analysis and interpretation of histological and ultrastructural data and in writing the manuscript. **S. Lepreux** (*Pathologist*) have made contribution to the histological, immunochemical, and ultrastructural studies. **M. Cario** (*Immune‐chemistry*, *Electron microscopy*) was involved in the examination and analysis of immunochemistry and electron microscopy, data analysis, and in the writing of manuscript. **J Rambert** (*Electron microscopy*) was involved in the examination of sections for electron microscopy. **M. Lafon** (*Virologist*) was involved in critically revising the manuscript and in trials of PCR assessment in SV skin samples. **A. Dakdaki (** *Molecular Biology* **)** have made contribution to the immunochemical study. **R. Abouqal** (*Statistical Analysis*) was involved in statistical study of histological and immunochemical data. **L. Benzekri** (*Dermatologist*, *Statistical Analysis*) was involved in recruitment of patients, in performing biopsies and made substantial contributions to the conception and the writing of the manuscript. **Dr Y. Gauthier** and **Pr L. Benzekri** contributed equally to this article.

## FUNDING INFORMATION

None.

## CONFLICT OF INTEREST

The authors declare that they have no conflict of interest.

## PATIENT CONSENT STATEMENT

Informed consent was obtained from all individual participants.

## Supporting information


Figure S1

Figure S2

Figure S3

Figure S4

Figure S5

Table S1

## Data Availability

All data generated or analyzed during this study are included in this published article and its supplementary information files. The datasets used and/or analyzed during the current study are available from the corresponding author on reasonable request.
