# Supplementary material for: Varicella‐zoster virus in actively spreading segmental vitiligo skin: Pathological, immunochemical, and ultrastructural findings (a first and preliminary study)
Source: Pigment Cell Melanoma Res. 2022 Oct 9;36(1):78–85. doi: 10.1111/pcmr.13064 (PMC10092484; doi:10.1111/pcmr.13064)
Supplement: Supplementary file 1 — Figure S1 [file PCMR-36-78-s001.docx]

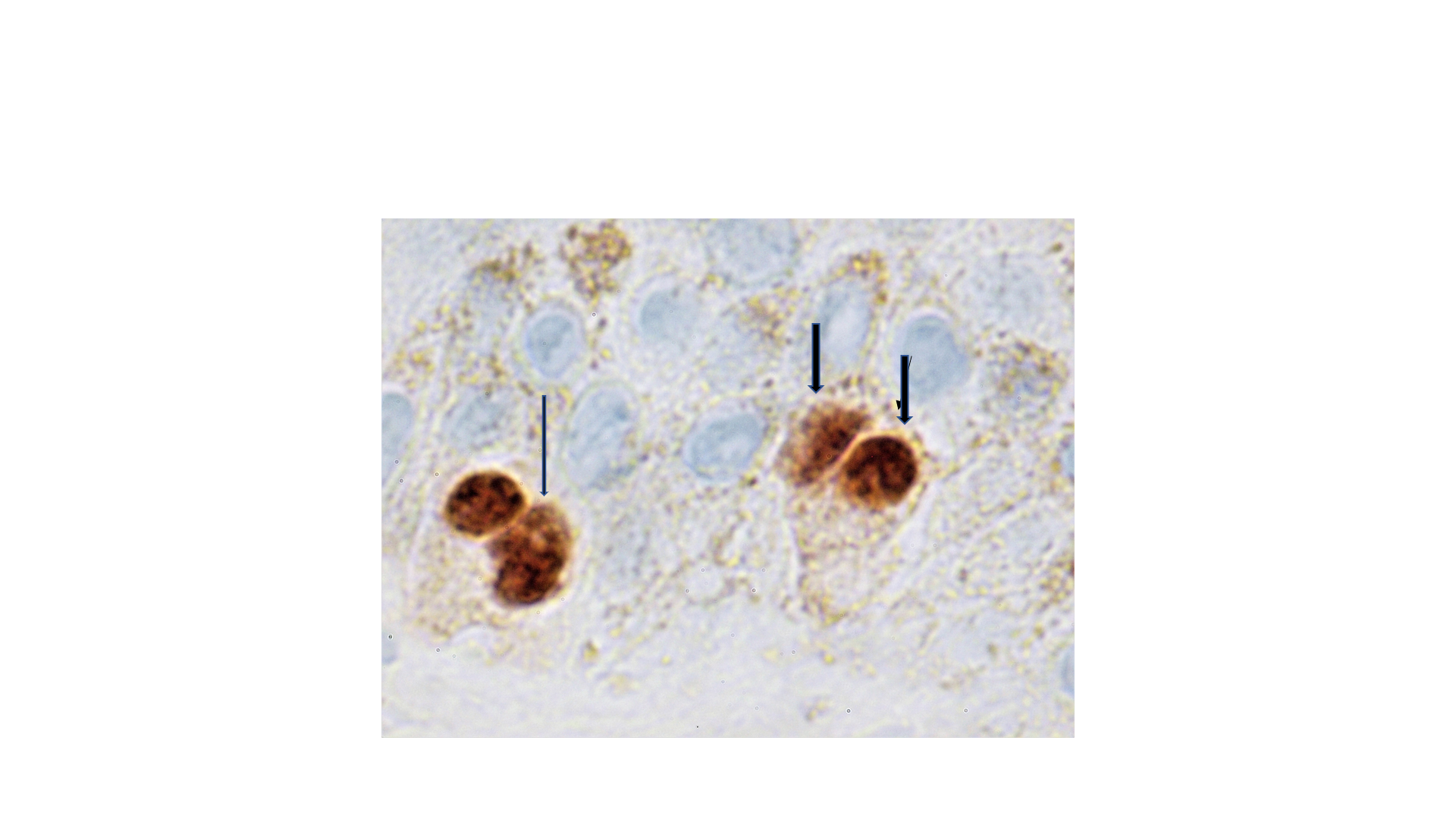


**SI Fig 1 : Viral cytopathic changes in marginal melanocytes of long lasting SV, left nucleus fusion ( black arrow), right cell fusion (black arrows)**

**MITFx 100**
