# Supplementary material for: Varicella‐zoster virus in actively spreading segmental vitiligo skin: Pathological, immunochemical, and ultrastructural findings (a first and preliminary study)
Source: Pigment Cell Melanoma Res. 2022 Oct 9;36(1):78–85. doi: 10.1111/pcmr.13064 (PMC10092484; doi:10.1111/pcmr.13064)
Supplement: Supplementary file 2 — Figure S2 [file PCMR-36-78-s004.docx]

**Supporting information Figure 2**


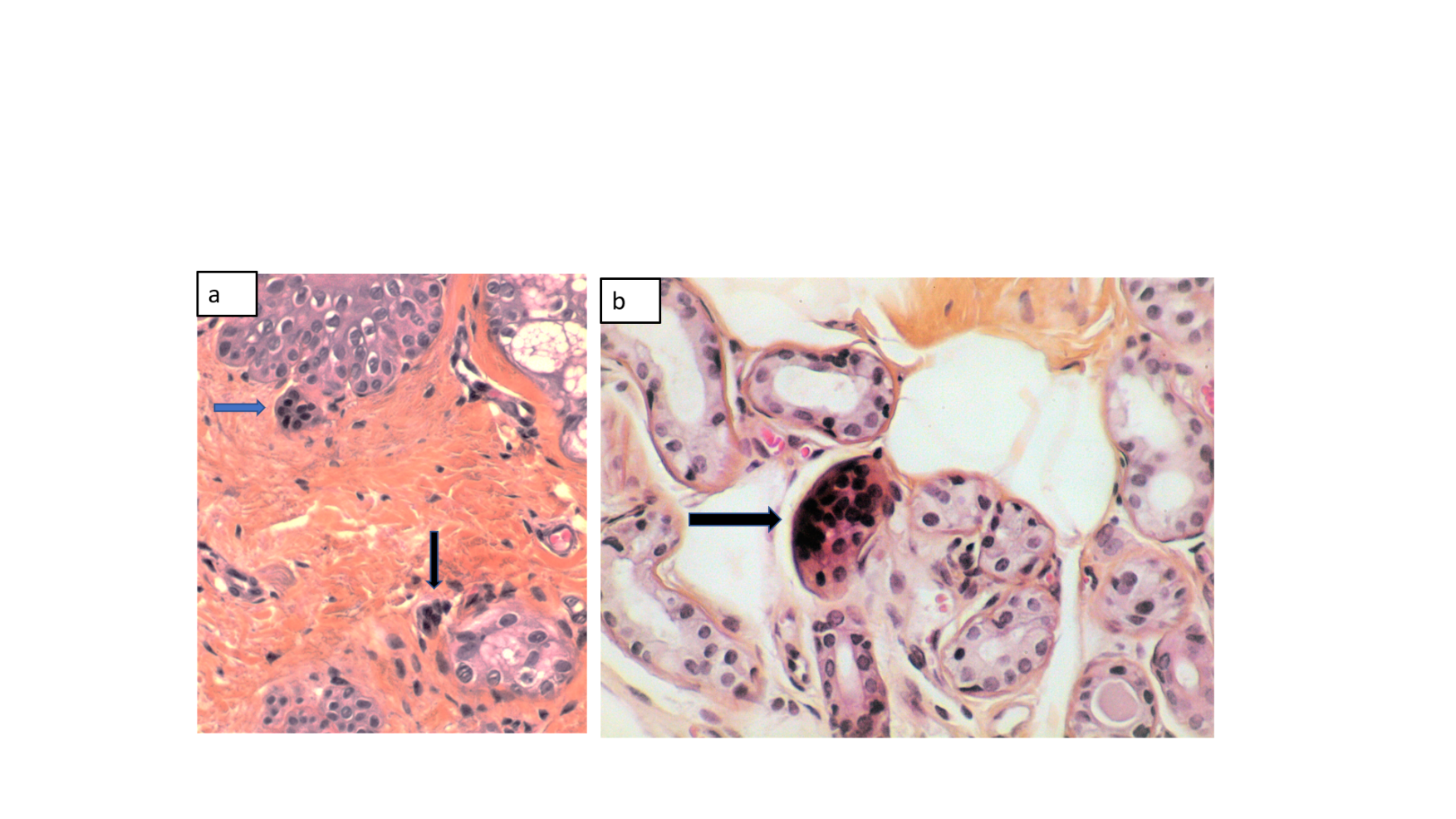


**SI Figure 2: Viral cytopathic changes in the dermis in long –lasting SV; (a) 2 pycnotic syncytia**

**( blue arrow and black arrows) HES x 20 ;(b) large syncytium (black arrow) in a sweat gland. HES x40.**
