# Supplementary material for: Varicella‐zoster virus in actively spreading segmental vitiligo skin: Pathological, immunochemical, and ultrastructural findings (a first and preliminary study)
Source: Pigment Cell Melanoma Res. 2022 Oct 9;36(1):78–85. doi: 10.1111/pcmr.13064 (PMC10092484; doi:10.1111/pcmr.13064)
Supplement: Supplementary file 3 — Figure S3 [file PCMR-36-78-s005.docx]

**Supporting information Figure 3**


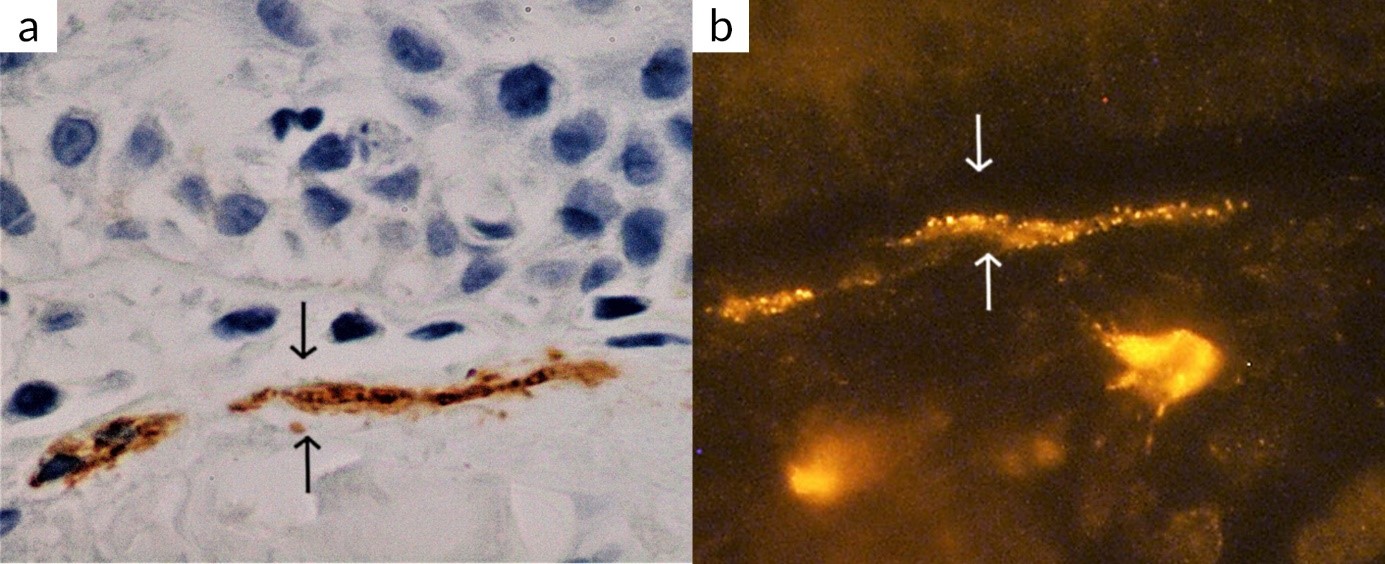


**SI figure 3 :a/ swelling (black arrows) of a superficial nerve (NGFR staining) x40, b/ VZV immunolabelling ( white arrow) of an other superficial nerve**

**(Immunofluorescence anti-VZV) x100**
