# Supplementary material for: Varicella‐zoster virus in actively spreading segmental vitiligo skin: Pathological, immunochemical, and ultrastructural findings (a first and preliminary study)
Source: Pigment Cell Melanoma Res. 2022 Oct 9;36(1):78–85. doi: 10.1111/pcmr.13064 (PMC10092484; doi:10.1111/pcmr.13064)
Supplement: Supplementary file 4 — Figure S4 [file PCMR-36-78-s002.docx]

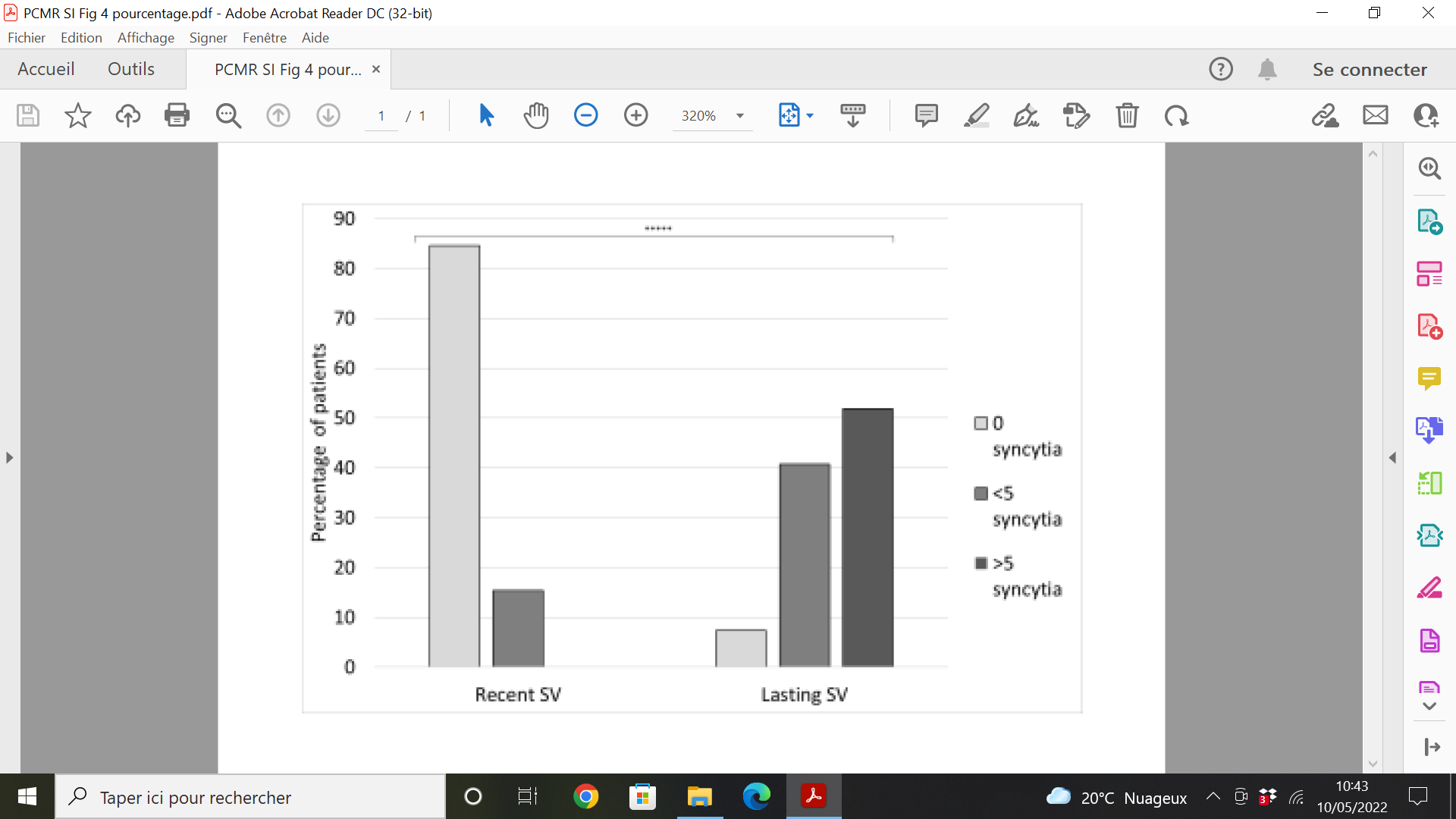


SI Figure 4 :**Percentage of SV patients with syncytia in**

**the dermis according to the age of SV lesions**
