# Supplementary material for: Varicella‐zoster virus in actively spreading segmental vitiligo skin: Pathological, immunochemical, and ultrastructural findings (a first and preliminary study)
Source: Pigment Cell Melanoma Res. 2022 Oct 9;36(1):78–85. doi: 10.1111/pcmr.13064 (PMC10092484; doi:10.1111/pcmr.13064)
Supplement: Supplementary file 5 — Figure S5 [file PCMR-36-78-s006.docx]

**Supporting information 5**


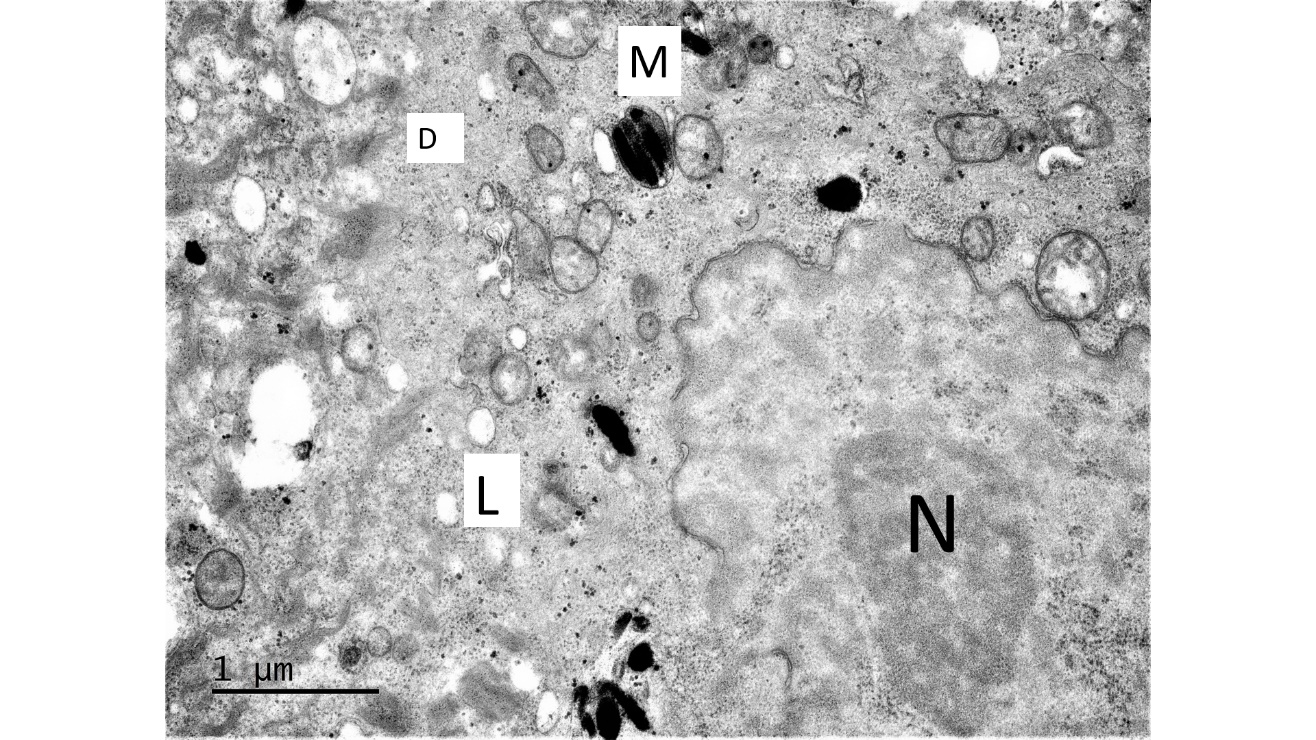


SI Fig 5: In SV, a basal keratinocyte with many “lysosomes like” **L** and few melanosomes **M** in its cytoplasm. **N: nucleus, D: desmosomes**
