# Supplementary material for: Varicella‐zoster virus in actively spreading segmental vitiligo skin: Pathological, immunochemical, and ultrastructural findings (a first and preliminary study)
Source: Pigment Cell Melanoma Res. 2022 Oct 9;36(1):78–85. doi: 10.1111/pcmr.13064 (PMC10092484; doi:10.1111/pcmr.13064)
Supplement: Supplementary file 6 — Table S1 [file PCMR-36-78-s003.docx]

S.I Table 1 : **VZV IgG antibodies titer in the serum of patients with SV**

| Technique | Number of SV | Positivity | Titer ranges | Median titer | Quartiles |
| --- | --- | --- | --- | --- | --- |
| **C.L.I.A** | 20 | > 175 | 554--3174 | 943 | 651.25 ;1676 |
| **E.L.I.S.A** | 11 | > 1.2 | 2.6--10 | 4.5 | 2.75 ;7 |

**Positivity**= Reference range required for positivity

Abbreviations : **C.L.J.A**= **Chemiluminescence Immunoassay (CLIA), E.L.I.S.A=** **Enzyme-Linked Immunosorbent Assay**
